# Acidification is required for calcium and magnesium concentration measurements in equine urine

**DOI:** 10.1186/s12917-023-03848-1

**Published:** 2024-01-10

**Authors:** Sandra Lapsina, Martina Stirn, Regina Hofmann-Lehmann, Angelika Schoster, Barbara Riond

**Affiliations:** 1 Clinical Laboratory, Department for Clinical Diagnostics and Services, Vetsuisse Faculty, University of Zurich, Zurich, Switzerland; 2 Clinic for Equine Medicine, Vetsuisse Faculty, University of Zurich, Zurich, Switzerland

**Keywords:** Phosphate, Fractional excretion of calcium and magnesium, Hydrochloric acid

## Abstract

**Background:**

Acidification of equine urine to promote dissociation of ion complexes is a common practice for urine ion concentration measurements. The objective of this study was to evaluate the effect of acidification and storage after acidification on calcium (Ca), magnesium (Mg) and phosphate (P) concentrations and on fractional excretion (FE) of these electrolytes. Thirty-two fresh equine urine samples were analysed between December 2016 and July 2020. Complete urinalysis (stick and sediment) was performed on all samples. Ca, Mg, P and creatinine concentrations were measured in supernatant of centrifuged native urine, urine directly centrifuged after acidification and urine centrifuged 1 hour after acidification. Urine was acidified with hydrochloric acid to reach a pH of 1–2. Ca, Mg, P and creatinine concentrations were also measured in blood plasma, and fractional excretion of each electrolyte was calculated. Equality of medians was tested with Friedman tests and Bland-Altman bias plots were used to show the agreement between conditions.

**Results:**

Acidification had a statistically significant effect on Ca and Mg concentrations, FE_Ca_ and FE_Mg_. Bland-Altman plot revealed a strong positive proportional bias between Ca concentration in native and acidified urine with a mean bias of 17.6 mmol/l. For Mg concentration, the difference between native and acidified urine was small with a mean bias of 1.8 mmol/l. The increase in FE_Ca_ was clinically relevant. Storage of acidified urine had no effect on any of the measured ion concentrations. All P concentrations in native urine samples were below the detection limit of the assay and statistical analysis and calculation of FE_P_ was not possible.

**Conclusions:**

Urine acidification is essential for accurate measurement of Ca and Mg concentrations and therefore FE calculations in equine urine. Storage time of 1 hour after acidification does not significantly change Ca and Mg concentrations.

**Supplementary Information:**

The online version contains supplementary material available at 10.1186/s12917-023-03848-1.

## Background

While the serum concentrations of Ca, Mg and P are part of a routine diagnostic bloodwork in horses, it is less well known that the urinary excretion measurements of these electrolytes can also serve diagnostic purposes [1, 2]. Physiologically, horses absorb up to 75% of dietary Ca, while adult humans, dogs, sheep and other domesticated animal species absorb only up to 55% of dietary Ca [3–6]. The main feed of horses is grass or hay which contains large amounts of Ca. However, horses also excrete rather large amounts of the absorbed Ca (approximating 30%) via kidneys, and their physiologically alkaline urine of pH 7.9 usually contains different amounts of crystals of which the most common are: Ca carbonate (CaCO_3_), Ca oxalate (CaC_2_O_4_·(H2O)_x_), triple phosphate (NH_4_MgPO_4_·6H_2_O) and Ca phosphate (Ca_3_ (PO_4_)_2_) [7–9]. The common urine crystals in horses contain Ca, Mg and P. The proportional distribution of each crystal type has not been studied in horses.

Since the concentration of each electrolyte in urine is influenced by the changes in water excretion rate, calculation of urinary fractional excretion (FE) for each electrolyte is considered more accurate [7, 10]. Fractional excretion of calcium in horses (FE_Ca_) varies between 7 to 33% depending on the breed, level of activity and diet of the horse [11]. Increased FE_Ca_ can indicate acute renal disease, as well as a Ca rich diet, while decreased values correspondingly have been associated with a diet low in Ca concentration and can also be observed in case of chronic renal failure [7, 12]. Fractional excretion of magnesium (FE_Mg_) usually parallels FE_Ca_ and varies between 15 to 53% with documented decreased values in case of chronic myositis [7, 11, 13]. FE_P_ in horses with adequate diet has been documented to be between 0 and 0.5 and < 4%, increased values being associated with diets high in P concentration, as well as such conditions as rhabdomyolysis, primary and pseudohyperthyroidism, nutritional secondary hyperparathyroidism, renal tubular disease and *P*-wasting nephropathy [14–17].

In human medicine the preanalytical acidification of urine samples has been used to dissolve Ca, Mg and P precipitates and thus obtain more accurate measurements of the said electrolyte concentrations. However, the acidification itself is a topic of divided opinion in human medicine: there are studies demonstrating its necessity [18–20] while some other studies claim this procedure unnecessary [21–24].

In veterinary diagnostics, the preanalytical acidification of equine urine samples prior to Ca, Mg and P measurement even though being common practice, to the authors` knowledge, has not been evaluated for its necessity. Nevertheless, there are some authors emphasizing inaccurate FE_Ca_ measurements without acidification [10].

We hypothesized that to accurately measure the concentration of common urine crystal components, such as Ca, Mg and P, the crystals need to be dissolved first. We wanted to assess what effect 1) acidification and 2) storage after acidification has on the Ca, Mg and P concentration measurements in equine urine and in turn on FE_Ca_ and FE_Mg_.

## Results

### Urinalysis

Complete urinalysis was available for 21/32 samples, while from 11 urine samples reagent strip analysis was not made. Results from urine specific gravity, reagent strip analysis and urine sediment examination are displayed in Supplementary file 1.

### Electrolytes and creatinine

pH, amount of crystals, Ca and Mg concentrations and FE_Ca_ and FE_Mg_ are displayed in Supplementary file 2 for each sample. The minimum, median and maximum values of Ca, Mg and creatinine (Crea) concentrations in the groups of native and acidified urine samples with and without storage are displayed in Table 1. The measurements of the acidified samples are corrected for the dilution factor for all parameters.

**Table 1:** Minimum, median and maximum values for Ca, Mg and Crea concentrations and fractional excretions of calcium and magnesium

Parameter	Minimum value	Median value	Maximum value
Ca native (mmol/L)	0.87	3.57	31.07
Ca acidified (mmol/L)* ^vs Ca native^	0.60	16.98	67.38
Ca acidified stored (mmol/L)*^vs Ca native^	0.56	16.98	66.38
Mg native (mmol/L)	1.33	16.01	35.59
Mg acidified (mmol/L)*^vs Mg native^	1.40	18.21	44.12
Mg acidified stored (mmol/L)**^vs Mg native^	1.30	18.35	44.35
Crea native (mmol/L)	1.03	12.43	28.57
Crea acidified (mmol/L) ^n.s. vs Crea native^	0.99	13.49	28.92
Crea acidified stored (mmol/L) ^n.s. vs Crea native^	0.98	13.71	27.52
FE_Ca_ native (%)	0.24	1.42	15.20
FE_Ca_ acidified (%)*** ^vs FE^_Ca_^native^	0.13	3.98	29.03
FE_Mg_ native (%)	2.94	21.67	44.26
FE_Mg_ acidified (%)**** ^vs FE^_Mg_^native^	2.82	21.52	45.85

*Ca* calcium, *Crea* creatinine**,** *FE*_*Ca*_ fractional excretion of calcium**,** *FE*_*Mg*_ fractional excretion of magnesium**,** *Mg* magnesium**,** *p < .0001**,** **p = 0.0015**,** ***p = 0.0003**,** ****p = 0.0039

**Fig. 1:**
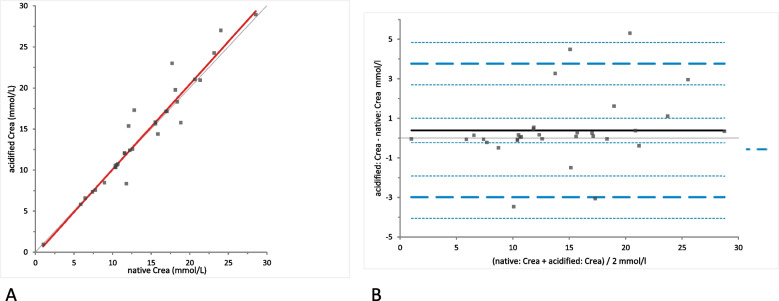
Comparison of the Crea (creatinine) concentration in native and acidified urine samples.** A** Passing Bablok regression plot for Crea concentration in native urine (x-axis) and acidified urine (y-axis). The thin grey line is the line of identity (y = x) and the thick red line is the line of best fit. The values after acidification were already corrected for the dilution factor. **B** Bland-Altman difference plot for the agreement of the Crea concentration measurements in native and acidified urine samples. The thin horizontal grey line (0 at the y-axis) is the line of identity, and the thick black line indicates the bias (mean difference between methods), with its confidence intervals as thin dashed lines. The thick dashed horizontal lines are the 95% limits of agreement with their 95% confidence intervals as the thin dashed lines. The mean difference is 0.39 (− 0.24 to 1.01)*, the Lower Limit of Agreement is − 2.99 (− 4.06 to − 1.92)*, the Upper Limit of Agreement is 3.76 (2.69 to 4.83)*

**Fig. 2:**
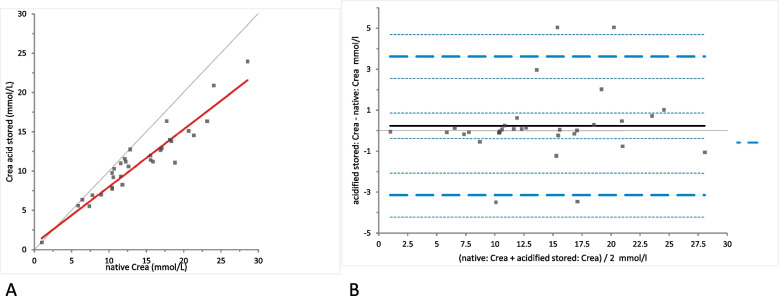
Comparison of the Crea (creatinine) concentration in native and acidified urine samples with storage. **A** Passing Bablok regression plot for Crea concentration in native urine (x-axis) and acidified urine after storage (y-axis). The thin grey line is the line of identity (y = x) and the thick red line is the line of best fit (the values after acidification are already corrected for the dilution factor). **B** Bland-Altman difference plot showing agreement of the Crea concentration measurements in native and acidified urine samples. The thin horizontal grey line (0 at the y-axis) is the line of identity, and the thick black line indicates the bias (mean difference between methods), with its confidence intervals as thin dashed lines. The thick dashed horizontal lines are the 95% limits of agreement with their 95% confidence intervals as the thin dashed lines. The mean difference is 0.23 (− 0.39 to 0.86)*, the Lower Limit of Agreement is − 3.14 (− 4.21 to − 2.07)*, the Upper Limit of Agreement is 3.608 (2.54 to 4.68)*

**Fig. 3:**
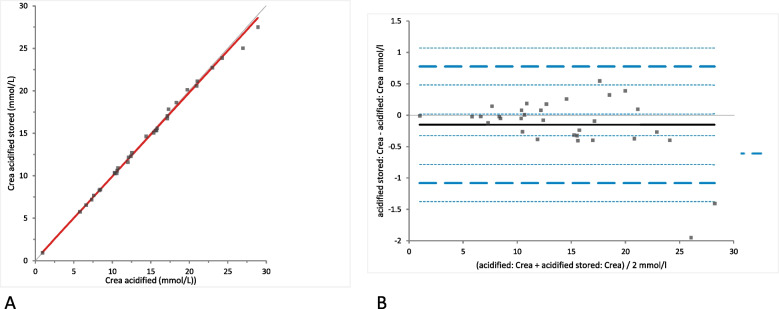
Comparison of the Crea (creatinine) concentration in acidified urine samples with and without storage. **A** Passing Bablok regression plot for Crea concentration in acidified urine (x-axis) and acidified urine after storage (y-axis). The thin grey line is the line of identity (y = x) and the thick red line is the line of best fit (the values after acidification are already corrected for the dilution factor). **B** Bland-Altman difference plot showing agreement of the Crea concentration measurements in native and acidified urine samples. The thin horizontal grey line (0 at the y-axis) is the line of identity, and the thick black line indicates the bias (mean difference between methods), with its confidence intervals as thin dashed lines. The thick dashed horizontal lines are the 95% limits of agreement with their 95% confidence intervals as the thin dashed lines. The mean difference is − 0.15 (− 0.32 to 0.02)*, the Lower Limit of Agreement is − 1.08 (− 1.37 to − 0.78)*, the Upper Limit of Agreement is 0.78 (0.48 to 1.07)*

Crea concentrations were not significantly different between the samples of native and acidified urine either without storage (mean difference 0.39 [LoA − 3.00 to 3.76], (*p*-value = 0.29)) (Fig. 1) or with storage (mean difference 0.23 mmol/L [LoA − 3.40 to 4.68], (*p*-value = 0.72)) (Fig. 2). Moreover, Crea concentrations did not change significantly in the acidified samples with storage (mean difference − 0.15 [LoA − 1.08 to 0.78], (*p*-value = 0.077)) (Fig. 3). Bland-Altman difference plots showed very small biases with random distribution without a systematic error (Figs. 1, 2 and 3B).

**Fig. 4:**
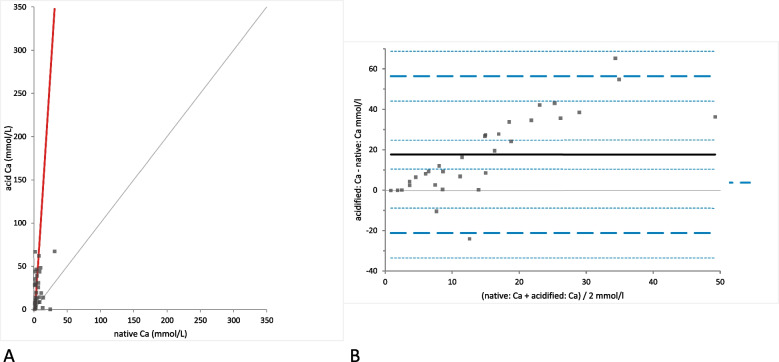
Comparison of the Ca (calcium) concentration in native and acidified urine samples.** A** Passing Bablok regression plot for Ca concentration in native urine (x-axis) and acidified urine (y-axis). The thin grey line is the line of identity (y = x) and the thick red line is the line of best fit (the values after acidification are already corrected for the dilution factor). **B** Bland-Altman difference plot showing agreement of the Ca concentration measurements in native and acidified urine samples. The thin horizontal grey line (0 at the y-axis) is the line of identity, and the thick black line indicates the bias (mean difference between methods), with its confidence intervals as thin dashed lines. The thick dashed horizontal lines are the 95% limits of agreement with their 95% confidence intervals as the thin dashed lines. The mean difference is 17.60 (10.47 to 24.73)*, the Lower Limit of Agreement is − 21.16 (− 33.47 to − 8.84)*, the Upper Limit of Agreement is 56.36 (44.05 to 68.67)*

**Fig. 5:**
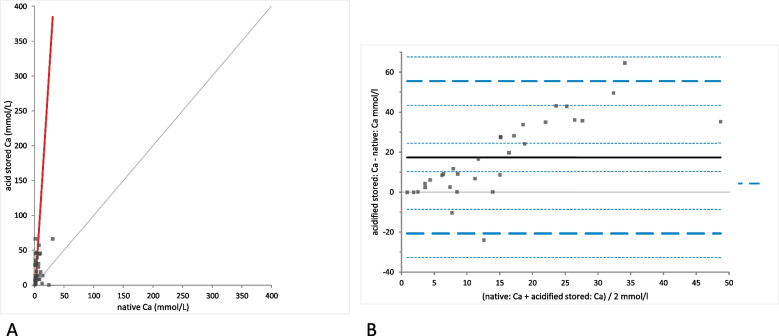
Comparison of the Ca (calcium) concentration in native and acidified urine samples with storage. **A** Passing Bablok regression plot for Ca concentration in native urine (x-axis) and acidified urine after storage (y-axis). The thin grey line is the line of identity (y = x) and the thick red line is the line of best fit (the values after acidification are already corrected for the dilution factor). **B** Bland-Altman difference plot showing agreement of the Ca concentration measurements in native and acidified urine samples. The thin horizontal grey line (0 at the y-axis) is the line of identity, and the thick black line indicates the bias (mean difference between methods), with its confidence intervals as thin dashed lines. The thick dashed horizontal lines are the 95% limits of agreement with their 95% confidence intervals as the thin dashed lines. The mean difference is 17.37 (10.37 to 24.36)*, the Lower Limit of Agreement is − 20.67 (− 32.75 to − 8.59)*, the Upper Limit of Agreement is 55.40 (43.32 to 67.48)*

**Fig. 6:**
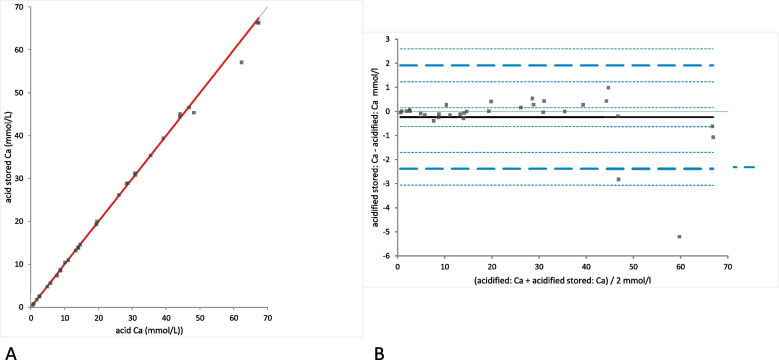
Comparison of the Ca (calcium) concentration in acidified urine samples with and without storage. **A** Passing Bablok regression plot for Ca concentration in acidified urine (x-axis) and acidified urine after storage (y-axis). The thin grey line is the line of identity (y = x) and the thick red line is the line of best fit (the values after acidification are already corrected for the dilution factor). **B** Bland-Altman difference plot showing agreement of the Ca concentration measurements in native and acidified urine samples. The thin horizontal grey line (0 at the y-axis) is the line of identity, and the thick black line indicates the bias (mean difference between methods), with its confidence intervals as thin dashed lines. The thick dashed horizontal lines are the 95% limits of agreement with their 95% confidence intervals as the thin dashed lines. The mean difference is − 0.23 (− 0.63 to 0.16)*, the Lower Limit of Agreement is − 2.38 (− 3.06 to − 1.70)*, the Upper Limit of Agreement is 1.91 (1.23 to 2.59)*

In contrast, Ca concentrations were significantly different in native urine samples when compared to acidified samples (mean difference 17.60 mmol/L [LoA − 21.16 to 56.36]; *p*-valued: < 0.0001; Fig. 4). This was also the case for stored samples where Ca concentrations were afterwards determined in native and acidified samples (mean difference 17.37 mmol/L [LoA − 20.67 to 55.40], *p*-value < 0.0001; Fig. 5). In both cases a strong positive proportional bias was observed in the Bland-Altman difference plot (Figs. 4B and 5B). No statistically significant change in Ca concentration was found in acidified samples due to storage (*p*-value = 0.72; Fig. 6A). The Bland-Altman difference plot showed a very small bias (mean difference − 0.23 mmol/L [LoA − 2.38 to 1.91]), with random distribution without a systematic error (Fig. 6B).

**Fig. 7:**
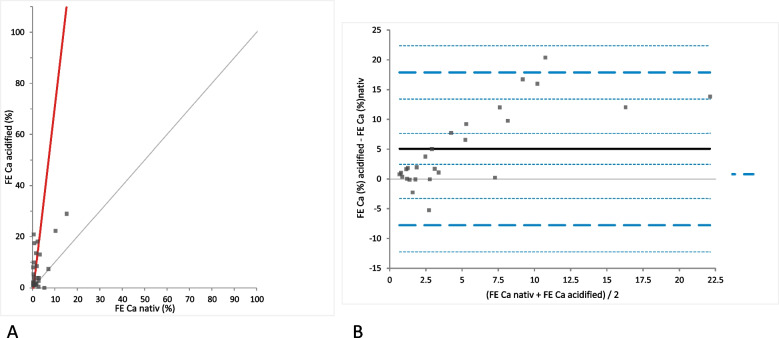
Comparison of the FE_Ca_ (fractional excretion of calcium) in native and acidified urine samples without storage. **A** Passing Bablok regression plot for FE_Ca_ (%) in native urine (x-axis) and FE_Ca_ in acidified urine (y-axis). The thin grey line is the line of identity (y = x) and the thick red line is the line of best fit (the values after acidification are already corrected for the dilution factor). **B** Bland-Altman difference plot showing agreement of the FE_Ca_ calculations in native and acidified urine samples without storage. The thin horizontal grey line (0 at the y-axis) is the line of identity, and the thick black line indicates the bias (mean difference between methods), with its confidence intervals as thin dashed lines. The thick dashed horizontal lines are the 95% limits of agreement with their 95% confidence intervals as the thin dashed lines. The mean difference is 5.07 (2.48 to 7.65)*, the Lower Limit of Agreement is − 7.74 (− 12.21 to − 3.27)*, the Upper Limit of Agreement is 17.88 (13.40 to 22.35)*

FE_Ca_ was calculated for both native and acidified urine samples without storage to better assess the Ca excretion. Calculations were possible only in 27/32 samples, since for the remaining 5 horses plasma was not available and, thus, for the equation needed blood plasma Ca and Crea concentrations could not be obtained. The FE_Ca_ significantly increased with acidification (mean difference 5.07% [LoA − 7.74 to 17.88]; *p*-value = 0.0003) with a positive proportional mean bias observed in the Bland-Altman difference plot (Fig. 7). In 3/27 of the native urine samples and in 11/27 of the acidified urine samples without storage the FE_Ca_ was within reference intervals (7 to 33%). 24/27 samples of the native urine and 16/27 acidified urine samples showed FE_Ca_ below the lower limit of the reference interval. In none of the groups increased values FE_Ca_ for were found.

**Fig. 8:**
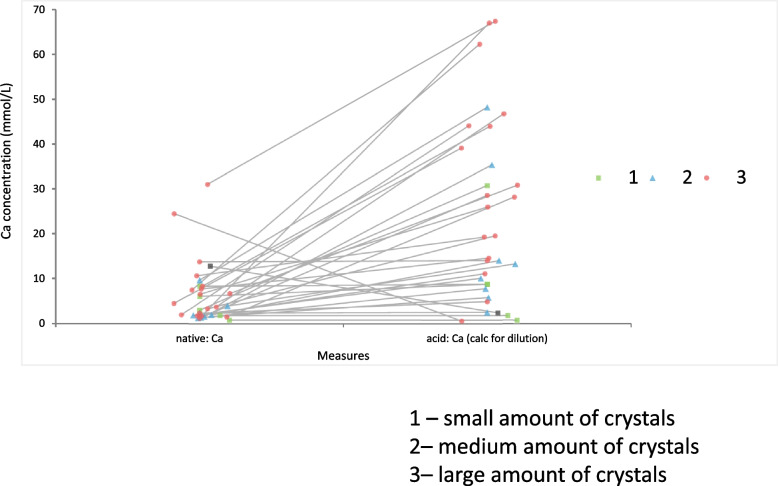
Depiction of the Ca (calcium) concentration in native and acidified urine samples correlated with semi-quantitative urine sediment crystal assessment. Each individual calcium measurement is depicted in both groups, connecting the results of the same sample with a grey line and labelling them with certain geometric shape and color according to the amounts of crystals observed in native sample: green rectangle – small amount of crystals, blue triangle – medium amount of crystals and red circle – large amount of crystals (the values after acidification are already corrected for the dilution factor)

Semiquantitative assessment of the quantity of crystals in the urine sediment revealed 4 samples (12.5%) with small amounts of crystals, 5 samples (15.6%) with medium amounts and 23 samples (71.9%) with large amounts of crystals. Moderate correlation (r_S_ = 0.42, *p* = 0.008) was observed between the amount of crystals seen in the sediment of the native samples and the acidified samples with significantly higher Ca concentration (Fig. 8). While in most cases the increase of the Ca concentration after acidification was proportional to the noted crystal amount in the native sample, several samples did not follow this pattern: 5 samples with large amounts of crystals showed lower Ca concentration after acidification than a sample with medium amounts of crystals; there were also samples where the Ca concentration after acidification hardly changed or even decreased, including two samples with large amounts of crystals.

**Fig. 9:**
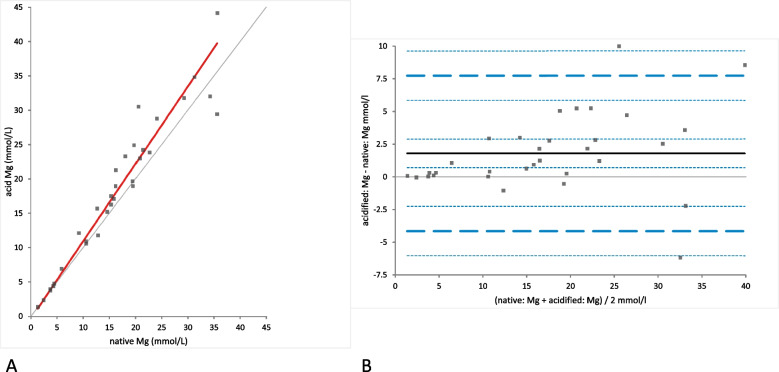
Comparison of the Mg (magnesium) concentration in native and acidified urine samples. **A** Passing Bablok regression plot for Mg concentration in native urine (x-axis) and acidified urine (y-axis). The thin grey line is the line of identity (y = x) and the thick red line is the line of best fit (the values after acidification are already corrected for the dilution factor). **B** Bland-Altman difference plot showing agreement of the Mg concentration measurements in native and acidified urine samples. The thin horizontal grey line (0 at the y-axis) is the line of identity, and the thick black line indicates the bias (mean difference between methods), with its confidence intervals as thin dashed lines. The thick dashed horizontal lines are the 95% limits of agreement with their 95% confidence intervals as the thin dashed lines. The mean difference is 1.79 (0.70 to 2.88)*, the Lower Limit of Agreement is − 4.14 (− 6.03 to − 2.26)*, the Upper Limit of Agreement is 7.72 (5.83 to 9.60)*

**Fig. 10:**
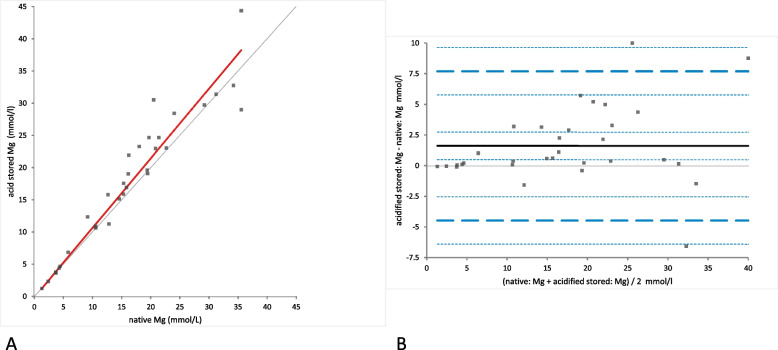
Comparison of the Mg (magnesium) concentration in native and acidified urine samples with storage. **A** Passing Bablok regression plot for Mg concentration in native urine (x-axis) and acidified urine samples after storage (y-axis). The thin grey line is the line of identity (y = x) and the thick red line is the line of best fit (the values after acidification are already corrected for the dilution factor). **B** Bland-Altman difference plot showing agreement of the Mg concentration measurements in native and acidified urine samples. The thin horizontal grey line (0 at the y-axis) is the line of identity, and the thick black line indicates the bias (mean difference between methods), with its confidence intervals as thin dashed lines. The thick dashed horizontal lines are the 95% limits of agreement with their 95% confidence intervals as the thin dashed lines. The mean difference is 1.62 (0.50 to 2.74)*, the Lower Limit of Agreement is − 4.46 (− 6.39 to − 2.53)*, the Upper Limit of Agreement is 7.70 (5.77 to 9.63)*

**Fig. 11:**
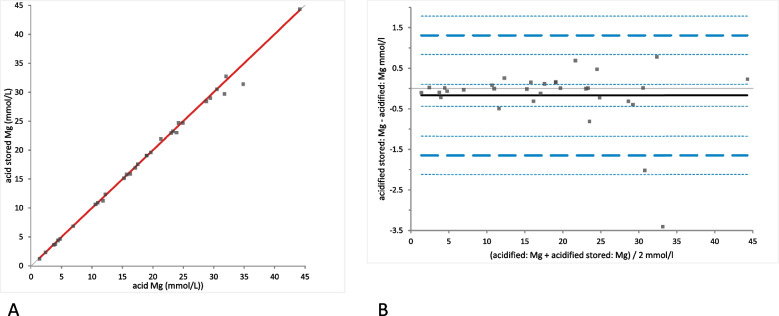
Comparison of the Mg (magnesium) concentration in acidified urine samples with and without storage. **A** Passing Bablok regression plot for Mg concentration in acidified urine samples (x-axis) and acidified urine samples after storage (y-axis). The thin grey line is the line of identity (y = x) and the thick red line is the line of best fit (the values after acidification are already corrected for the dilution factor). **B** Bland-Altman difference plot showing agreement of the Mg concentration measurements in native and acidified urine samples. The thin horizontal grey line (0 at the y-axis) is the line of identity, and the thick black line indicates the bias (mean difference between methods), with its confidence intervals as thin dashed lines. The thick dashed horizontal lines are the 95% limits of agreement with their 95% confidence intervals as the thin dashed lines. The mean difference is − 0.17 (− 0.44 to 0.10)*, the Lower Limit of Agreement is − 1.64 (− 2.11 to − 1.17)*, the Upper Limit of Agreement is 1.31 (0.84 to 1.78)*

Mg concentration was significantly different between native and acidified urine samples determined either without storage **(**mean difference 1.79 mmol/L [LoA from − 4.14 to 7.72], (*p*-value = 0.0001)) (Fig. 9A) or with storage (mean difference 1.62 mmol/L [LoA from − 4.46 to 7.70], (*p*-value = 0.0015)) (Fig. 10A). In the Bland-Altman difference plots a small bias with random distribution without systematic difference was observed (Figs. 9B and 10B). Similar to Ca concentrations, no significant difference was found between the Mg concentrations in acidified urine with and without storage (mean difference − 0.17 mmol/L [LoA from − 1.64 to 1.78]); (*p*-value = 1.00); (Fig. 11A). Bland-Altman difference plot showed very small bias with random distribution without systematic difference (Fig. 11B).

**Fig. 12:**
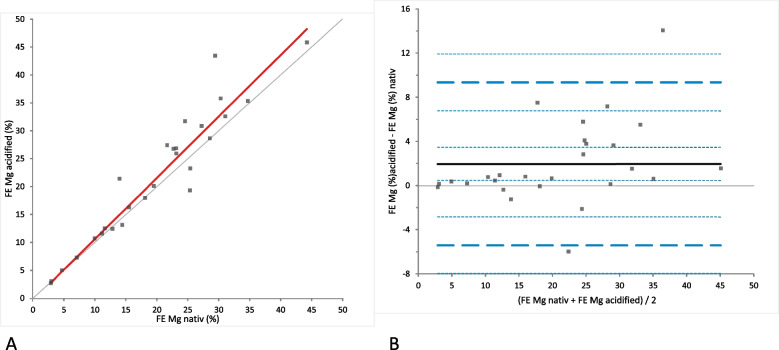
Comparison of the FE_Mg_ (fractional excretion of magnesium) in native and acidified urine samples without storage. **A** Passing Bablok regression plot for FE_Mg_ in native urine samples (x-axis) and acidified urine samples(y-axis). The thin grey line is the line of identity (y = x) and the thick red line is the line of best fit (the values after acidification are already corrected for the dilution factor). **B** Bland-Altman difference plot showing agreement of the FE_Mg_ calculations in native and acidified urine samples without storage. The thin horizontal grey line (0 at the y-axis) is the line of identity, and the thick black line indicates the bias (mean difference between methods), with its confidence intervals as thin dashed lines. The thick dashed horizontal lines are the 95% limits of agreement with their 95% confidence intervals as the thin dashed lines. The mean difference is 1.98 (0.49 to 3.47)*, the Lower Limit of Agreement is − 5.39 (− 7.97 to − 2.82)*, the Upper Limit of Agreement is 9.35 (6.78 to 11.92)*. Abbreviations: Ca = calcium; Mg = magnesium; *P* = phosphate; HCL = hydrochloric acid. *Numbers in parentheses are 95% confidence intervals

FE_Mg_ was calculated in both native and acidified urine without storage. In 27/32 samples these calculations were possible, while this was not possible for the remaining 5 horses due to unavailability of the corresponding blood plasma Mg and Crea concentrations. There was a significant difference between FE_Mg_ using native compared to acidified urine samples (Fig. 12A). Bland-Altmann difference plot showed a small bias with random distribution without systematic difference (mean difference 1.98 [LoA from − 5.39 to 9.35]), (*p*-value = 0.0039)) (Fig. 12B). In 17/27 of the native urine samples and in 18/27 of the acidified urine samples without storage the FE_Mg_ was within the reference intervals (15 to 53%). In 10/27 of the native urine samples and in 9/27 of the acidified urine samples without storage the FE_Mg_ was below the reference intervals. In none of the groups increased values for FE_Mg_ were found.

For the measurements of the P concentration all native samples were under the lower limit of quantification of the assay (1.1 mmol/l). After acidification, 3 samples were mildly above the lower limit of quantification in the group without and with storage (values between 1.33–2.42 mmol/l. Statistical analysis was not possible due to the low number of samples.

## Discussion

This is the first study evaluating the effect of acidification and storage after acidification on Ca, Mg, P concentrations, FE_Ca_ and FE_Mg_ in equine urine from horses presented to the clinic at the University Animal Hospital Zurich. Our data demonstrated significantly higher Ca and Mg concentrations after urine acidification and in turn significantly higher FE of these electrolytes. P concentrations could not be evaluated as only 9% of all acidified and none of the native samples had a concentration above the detectable limit. One hour storage after acidification did not lead to significant changes in any electrolyte concentrations or FE.

The increase in Ca and Mg concentrations after acidification of urine with hydrochloric acid (HCI) can be explained by the dissolution of urine crystals, thereby enabling the measurement of the Ca and Mg ions previously confined in crystals. This assumption seems plausible since it is well known that the most commonly observed crystals in equine urine are Ca carbonate, Ca oxalate, triple phosphate and Ca phosphate and they all contain Ca, with the triple phosphates also containing Mg [7, 8, 25]. As demonstrated in previous studies, alkaline pH crystals such as Ca carbonate, Ca phosphate and hydroxyapatite are already dissolving at pH 5–6, while acidification to pH 1–2 should ensure complete dissolution [26–28]. Further evidence can be provided by the fact that the majority (12/19) of samples with large amounts of crystals showed an increase in Ca concentration in the acidified urine of ≥30%. However, statistical correlation between the amount of crystals and the increase in Ca concentration after acidification was only moderate, since some samples with moderate amounts of crystals showed higher Ca concentration after acidification than some samples with large amounts of crystals. Moreover, a few samples even showed a decrease in Ca concentration after acidification, including 2 samples with large amounts of crystals. Reasons for different reactions of different samples when subjected to acidification can be diverse. Different crystals are expected to yield different amounts of Ca ions when dissolved. Also, ultra-small crystals could have been present in the native urine sample, which would be observed in the nucleation phase of the crystal forming; they would unlikely be detected by light microscopy [29] but contribute to ion concentrations. Moreover, incomplete dissolution after acidification could not be ruled out because sediment analysis of the acidified sample was not performed. The growth and dissolution of crystals depends on pH which slightly fluctuates depending on the time of the day, feeding regime, diet and time of the year. According to one study, the urine pH is slightly lower during summer months, at 8 am and for horses fed with grain [9]. The samples for this study were collected during different times of the day and months of the year. Finally, errors in processing the urine samples, such as insufficient mixing of the samples resulting in an uneven distribution of crystals in the native and acidified sample as well as inter-observer variation during semi-quantitative assessment of the amount of crystals by different laboratory technicians need to be considered; the latter procedure is known to be a subjective procedure.

Within this study, also the effect of acidification on urine Crea concentration has been investigated since urine Crea concentration is needed for calculation of the FE. There was no difference between Crea concentrations from native and acidified equine urine samples. Crea concentration in the acidified samples was corrected for the dilution factor.

Storage of an acidified urine sample for 1 hour at room temperature had no significant impact on either Ca or Mg concentration measurements. The 1-hour storage was chosen to imitate delays in the processing of an acidified urine sample. Since the acidification procedure might be a problem for equine practitioners in the stable, further studies should be performed with longer storage periods to mimic over-night shipment of native and acidified urine to get more preanalytical data on storage of equine urine for electrolyte measurement.

Only 3 study samples showed P concentration values mildly above the lower limit of quantification of the used assay. Published reference intervals for FE_P_ are very low and, therefore our results match those in previous studies [10, 30]. Increased P concentration in urine as well as increased FE_P_ is a rather rare occurrence in horses and was also not present in our study population. To further evaluate the necessity of acidification for P measurement in equine urine, samples from horses with higher P concentrations are needed.

For FE_Ca,_ 24/27 samples showed FE values below the RI, and 3/27 samples had FE within RI in the native urine sample (RI 7–33%). After acidification, 8 samples shifted from decreased FE_Ca_ to normal FE_Ca_ values, whereas the remaining 16 samples had still decreased FE_Ca_. The three samples which showed already in native urine normal FE_Ca_ stayed within normal ranges even after acidification. The clinical interpretation of the FE_Ca_ values using published reference intervals (7–33%) differed therefore in 30% of all samples after acidification of the urine. Based on these observations it can be concluded that the difference in FE_Ca_ after acidification is not only statistically significant but also of clinical importance since measurement of native – non acidified urine samples could lead to misinterpretation.

For FE_Mg_, 10/27 native urine samples showed FE values below the RI, whereas 17/27 samples had FE within RI (15–53%). After acidification only 1 sample (4% of all samples) originally with decreased FE in the native sample showed FE within RI. In all other samples acidification did not change the interpretation outcome.

Based on this study, Ca concentration and FE_Ca_ were more affected than Mg and FE_Mg_ by acidification. This might be due to the higher amounts of Ca present in the crystals compared to Mg. Furthermore the wide RI for FE_Mg_ might impact the smaller clinical differences compared to FE_Ca_ .

Therefore, it can be stated, that for Ca and FE_Ca_ interpretation, acidification of equine urine prior to analysis is crucial and of high clinically relevance. For Mg and FE_Mg_ measurements, acidification is at least recommended, since statistically significant changes and clinical differences were observed within this study.

There were several limitations to this study. First, complete urinalysis could not be performed in some of the urine samples as data for pH, specific gravity and reagent strip were missing. Second, the present study population did not contain samples with FE_Ca_ and FE_Mg_ values above the upper reference interval. Therefore, only shifts from low FE results to unremarkable FE results could be observed after acidification. However, since reference limits for FE have a wide range for both Ca and Mg, one should give great attention to the observed shifts from one to the other category. Further investigations should be done with samples of increased FE after acidification. In addition, it would have been of interest to use ion selective potentiometry for Ca and Mg determination. Moreover, the study would be strengthened if the mechanism for acidification on increasing urine Ca and Mg concentration was explored further. Finally, incomplete dissolution of the crystals after acidification could not be ruled out as sediment analysis of the acidified samples was not performed.

## Conclusion

Urine acidification is necessary for accurate measurements of Ca and Mg concentrations and subsequent FE calculations in equine urine. Especially for Ca and FE_Ca_ interpretation, marked and clinically relevant differences between native and acidified samples were observed thus shifting interpretation from low concentrations to normal levels. Storage time of 1 hour after acidification does not significantly change Ca and Mg concentrations. It is important to consider that acidified urine samples are not adequate for urine reagent strip analysis, therefore urine needs to be analyzed in two tubes, one with and one without acidification, whenever Ca, Mg or FE measurements are required.

## Methods

### Materials

The study was conducted as a prospective study with samples collected between December 2016 and July 2020. In total 22 client-owned horses were involved in the study. The horses were presented at the Equine Department of the Vetsuisse Faculty of the University of Zurich for various reasons. Blood sampling was approved by the local animal welfare committee of the Veterinary Office, Canton Zurich, Switzerland (ZH042/15; ZH057/19). Furthermore, informed owner consent was obtained for all horses sampled within this study.

Four out of the 22 horses were sampled multiple times (three to five times). In total 32 fresh free-catch equine urine samples were collected in plain tubes (Sarstedt AG & Co., Nürmbrecht, Germany). Urine sampling and a corresponding blood sample from the jugular vein were collected from 17 horses in tri-potassium ethylenediamine tetraacetic acid (K_3_EDTA) and lithium heparin tubes. All samples were analysed by laboratory technicians within 2 hours after collection.

### Clinical blood biochemistry

Plasma concentrations of Crea, Ca, Mg, and P were determined in 17/22 horses at the first sampling of each horse in lithium heparin plasma according to the Clinical & Laboratory Standards Institute guidelines using the biochemistry analyser Cobas c501 (Roche Diagnostics, Rotkreuz, Switzerland) and reference intervals developed in the Clinical Laboratory of the Vetsuisse Faculty of the University of Zurich from 63 adult horses. Lithium heparin tubes were centrifuged for 5 minutes at 1862 g with Hettich Rotanta 460 s centrifuge (Hettich AG, Bäch, Switzerland) to collect heparin plasma. Internal QC was performed daily prior to processing the routine samples using 2 levels of the QC PreciControl ClinChem Multi (Roche Diagnostics, Mannheim, Germany to assess accuracy and precision of Crea, Ca, Mg and P measurements Level 1 had target concentrations within the lower concentration range whereas level 2 had target concentrations within the normal range of concentration.

### Urinalysis and urine clinical biochemistry measurements

From each horse, 15 ml of fresh native urine were used for the study. The urine was thoroughly mixed and then separated into two tubes – the first tube (5 ml) was used for the complete urinalysis as well as for the determination of Ca, Mg and P ion concentrations directly after sampling. The second tube (10 ml) was acidified with HCl (1 M) to pH 1–2 using a pipette to exactly measure the added HCI volume and pH indicator paper to document the pH value. This second acidified tube was immediately and equally further separated into two tubes, 5 ml each. The first tube was used for Ca, Mg and P concentration measurements directly after acidification, while the second one was stored at room temperature (+ 22^0^ C) for 1 hour before the measurements of Ca, Mg and P concentration. Ca, Mg and P ion concentration were determined on a Cobas C501 biochemistry analyser (Roche Diagnostics, Rotkreuz, Switzerland) using the following photometric methods: 5-Nitro-5′-methyl-BAPTA (NM-BAPTA) assay for Ca ion concentration, colorimetric end-point assay for Mg ion concentration and ammonium molybdate assay for P ion concentration. Internal QC was performed daily prior to processing the routine samples using 2 levels of the QC Lyphochek Quantitative Urine Control 1 and 2 (Bio-Rad Laboratories, Hercules, California, United States). The volume of HCl used to reach a pH of 1–2 was noted for each acidified sample and afterwards each measurement was corrected for the dilution factor. FE was calculated for each electrolyte using the equation:

$$FEx=\frac{\left[x\right] urine}{\left[x\right] serum}X\frac{Crea\ serum}{Crea\ urine}X\ 100$$

Where x = electrolyte under investigation.

[]urine = urinary concentration of the substance.

[]serum = serum concentration of the substance.

A complete urinalysis was performed according to the standard operating procedure of the laboratory, which included macroscopic examination, determination of urine specific gravity by refractometry using Atago URICON-NE refractometer (Atago, Tokyo, Japan), reagent strip analysis for protein, glucose, ketones, bilirubin and blood using Combur 10-Test M strips (Roche Diagnostics, Rotkreuz, Switzerland) and Cobas U 411 urine analyser (Roche Diagnostics, Rotkreuz, Switzerland). pH measurements were obtained by using pH-indicator paper (Merck Millipore, Billerica, USA). The Liquicheck Urinalysis Control (Bio-Rad Laboratories, Hercules, California, United States) was used daily as an internal quality control prior to daily urine sample analyses on Cobas U 411.

For the urine sediment assessment, 5 ml of the native urine sample were centrifuged for 5 minutes at 400 g using a Hettich Rotanta 460S centrifuge (Hettich, Kirchlengern, Germany). Afterwards the supernatant was poured off to be used for the quantification of Ca, Mg and P concentration. An unstained sediment microscopy was performed by a laboratory technician using a Leitz Dialux 20 microscope (Leica, Wetzlar, Germany) in phase-contrast and dark-field microscopy in 100x and 400x magnification. The type and amount of the observed crystals were noted and the samples were classified as containing either *small, medium* or *large* amounts of crystals.

#### Statistical analysis

The data was processed with MS Excel and Analyse-it® (Analyse-it Software, Leeds, United Kingdom). Friedman test was used to compare the medians of electrolytes and urine creatinine across groups. Passing-Bablok regression analysis and Bland-Altman difference plots were used to assess agreement between native and acidified urine samples. Statistical significance were defined as *p*-value < 0.05. Spearman’s rank correlation coefficient (r_S_) and descriptive statistics was made for correlation of amount of crystals and concentration of Ca after acidification without storage.

### Supplementary Information

**Additional file 1.**

**Additional file 2.**

## Data Availability

The authors confirm that the main data supporting the findings of this study are available within the article. The complete raw data are available from the corresponding author, Barbara Riond, upon reasonable request.

## Abbreviations

Ca: Calcium
Ca/Crea: Calcium/creatinine ratio
CI: Confidence intervals
Crea: Creatinine
FE: Fractional excretion
FE_Ca_: Fractional excretion of calcium
FE_Mg_: Fractional excretion of magnesium
FE_P_: Fractional excretion of phosphate
HCl: Hydrochloric acid
K_3_EDTA: Tri-potassium ethylenediamine tetraacetic acid
LoA: Limits of agreement
Mg: Magnesium
NM-BAPTA: 5-Nitro-5′-methyl-BAPTA
P: Phosphate
