# Supplementary material for: Acidification is required for calcium and magnesium concentration measurements in equine urine
Source: BMC Vet Res. 2024 Jan 10;20:21. doi: 10.1186/s12917-023-03848-1 (PMC10777620; doi:10.1186/s12917-023-03848-1)
Supplement: Supplementary file 1 — Additional file 1. [file 12917_2023_3848_MOESM1_ESM.docx]

*Supplementary file 1*

**The specific gravity, pH, chemical and sediment analysis of each urine sample**

| No. of the sample | Specific gravity (≥1.025) | Native urine pH (7.5-8.5) | reagent strip | | | | | sediment | | | | | |
| --- | --- | --- | --- | --- | --- | --- | --- | --- | --- | --- | --- | --- | --- |
|  |  |  | Protein (neg) | Glucose (neg) | Ketones (neg) | Bilirubin (neg) | Blood (neg) | Erythrocytes/ hpf (0-5) | Leucocytes/ hpf (0-5) | Ca carbonate crystals*  (various) | Ca oxalate crystals* (various) | Amorphous crystals* (Various) | Bacteria (N) |
| 1 | NA | 7.96 | NA | NA | NA | NA | NA | NA | NA | 3 | N | N | - |
| 2 | NA | 8.45 | NA | NA | NA | NA | NA | NA | NA | 3 | N | N | - |
| 3 | NA | 7.92 | NA | NA | NA | NA | NA | NA | NA | 3 | N | N | - |
| 4 | NA | 7.71 | NA | NA | NA | NA | NA | NA | NA | 3 | N | N | - |
| 5 | NA | 8.28 | NA | NA | NA | NA | NA | NA | NA | 2 | N | 3 | - |
| 6 | 1.014 | 8.00 | neg | neg | neg | neg | neg | N | N | 1 | 2 | N | N |
| 7 | 1.035 | 8.00 | 1+ | neg | neg | 1+ | neg | 0-4 | 0-4 | 3 | N | N | N |
| 8 | 1.036 | 8.00 | 2+ | neg | neg | 1+ | neg | 0-4 | 0-4 | 1 | N | 3 | N |
| 9 | 1.035 | 8.00 | 1+ | neg | neg | neg | neg | N | N | 3 | N | N | N |
| 10 | 1.035 | 7.50 | neg | neg | neg | 1+ | neg | 0-4 | 0-4 | 3 | N | N | N |
| 11 | 1.035 | 8.50 | 2+ | neg | neg | 1+ | neg | N | N | 3 | N | N | N |
| 12 | 1.031 | 8.00 | 1+ | neg | neg | 1+ | neg | N | N | 3 | N | N | N |
| 13 | NA | 8.00 | NA | NA | NA | NA | NA | N | N | 3 | N | N | N |
| 14 | 1.034 | 8.00 | 1+ | neg | neg | neg | neg | N | N | 3 | N | N | N |
| 15 | 1.031 | 8.00 | 1+ | neg | neg | 1+ | neg | N | N | 2 | N | N | rare |
| 16 | 1.027 | 8.00 | neg | neg | neg | neg | neg | N | N | 3 | N | N | N |
| 17 | NA | 8.50 | NA | NA | NA | NA | NA | NA | NA | 2 | N | N | N |
| 18 | 1.030 | 8.00 | neg | neg | neg | neg | neg | N | N | 3 | N | N | N |
| 19 | 1.032 | 8.50 | 2+ | neg | neg | neg | neg | N | N | 2 | N | N | few |
| 20 | 1.033 | 8.00 | 1+ | neg | neg | 1+ | neg | N | N | 2 | N | N | N |
| 21 | 1.027 | 8.00 | neg | neg | neg | neg | neg | 0-4 | 0-4 | 3 | N | N | N |
| 22 | 1.047 | 8.00 | 1+ | neg | neg | neg | neg | 4-8 | 0-4 | 3 | N | N | N |
| 23 | 1.017 | 7.00 | 2+ | neg | neg | neg | 5+ | 4-8 | 0-4 | N | 1 | 1 | N |
| 24 | 1.024 | 7.50 | 1+ | neg | neg | neg | 5+ | 0-4 | 0-4 | 3 | N | N | N |
| 25 | 1.025 | 8.00 | 1+ | neg | neg | neg | neg | N | N | 3 | N | N | N |
| 26 | 1.028 | 8.00 | 2+ | neg | neg | neg | neg | N | N | 3 | N | N | N |
| 27 | NA | 7.50 | NA | NA | NA | NA | NA | NA | NA | 1 | N | N | N |
| 28 | 1.014 | 7.50 | 2+ | neg | neg | neg | 1+ | 0-4 | N | 3 | 2 | N | N |
| 29 | NA | 7.50 | NA | NA | NA | NA | NA | NA | NA | 1 | N | N | N |
| 30 | NA | 8.00 | NA | NA | NA | NA | NA | NA | NA | 3 | N | N | N |
| 31 | NA | 7.00 | NA | NA | NA | NA | NA | NA | NA | 3 | N | N | N |
| 32 | 1.012 | 8.00 | 1+ | neg | neg | neg | neg | 4+ | 20-30 | 2 | N | N | rare |

Ca = calcium; N = not detected; neg = negative; NA = not available

*grading of the amount of crystals, 1 = few, 2 = some, 3 = many

() reference intervals for adult horses[30]
