# Supplementary material for: Acidification is required for calcium and magnesium concentration measurements in equine urine
Source: BMC Vet Res. 2024 Jan 10;20:21. doi: 10.1186/s12917-023-03848-1 (PMC10777620; doi:10.1186/s12917-023-03848-1)
Supplement: Supplementary file 2 — Additional file 2. [file 12917_2023_3848_MOESM2_ESM.docx]

*Table 2*

**Amount of crystals, as well as concentrations and fractional excretion of calcium and magnesium**

| No. of the sample | Semiquantitative amount of crystals in native urine sample* | Ca concentration mmol/l in native urine | Ca concentration mmol/l in acidified urine | Ca concentration mmol/l in acidified urine with storage | Mg concentration mmol/l in native urine | Mg concentration mmol/l in acidified urine | Mg concentration mmol/l in acidified urine with storage | FE_Ca_ in native urine | FE_Ca_ in acidified urine | FE_Mg_ in native urine | FE_Mg_ in acidified urine |
| --- | --- | --- | --- | --- | --- | --- | --- | --- | --- | --- | --- |
| 1 | 3 | 8.38 | 44.01 | 44.45 | 31.23 | 34.81 | 31.41 | - | - | - | - |
| 2 | 3 | 3.37 | 19.60 | 20.02 | 35.56 | 29.40 | 29.01 | - | - | - | - |
| 3 | 3 | 1.63 | 35.40 | 35.40 | 12.85 | 11.80 | 11.31 | - | - | - | - |
| 4 | 3 | 9.71 | 48.24 | 45.43 | 29.23 | 31.76 | 29.75 | - | - | - | - |
| 5 | 3 | 1.90 | 10.14 | 10.42 | 10.57 | 10.60 | 10.68 | - | - | - | - |
| 6 | 2 | 8.36 | 8.80 | 8.56 | 3.72 | 4.02 | 3.80 | 1.42 | 1.33 | 2.94 | 2.82 |
| 7 | 3 | 3.76 | 46.81 | 46.62 | 18.07 | 23.29 | 23.30 | 0.67 | 9.89 | 13.99 | 21.52 |
| 8 | 3 | 3.02 | 30.82 | 31.25 | 19.70 | 24.92 | 24.70 | 0.56 | 4.36 | 12.85 | 12.51 |
| 9 | 3 | 1.51 | 28.25 | 28.80 | 12.69 | 15.69 | 15.85 | 0.39 | 5.44 | 14.41 | 13.20 |
| 10 | 3 | 10.68 | 19.33 | 19.34 | 34.20 | 31.98 | 32.76 | 2.24 | 3.98 | 25.41 | 23.34 |
| 11 | 3 | 1.79 | 11.15 | 11.00 | 15.85 | 17.09 | 16.97 | 0.32 | 1.98 | 9.98 | 10.78 |
| 12 | 3 | 2.38 | 4.91 | 4.84 | 19.49 | 18.95 | 19.12 | 0.66 | 1.08 | 25.36 | 19.42 |
| 13 | 3 | 6.56 | 26.05 | 26.22 | 14.61 | 15.24 | 15.22 | 3.26 | 13.04 | 31.06 | 32.64 |
| 14 | 3 | 13.80 | 14.08 | 14.03 | 19.41 | 19.65 | 19.67 | 2.76 | 2.77 | 18.10 | 18.06 |
| 15 | 2 | 3.98 | 13.31 | 13.20 | 15.32 | 16.25 | 15.95 | 0.86 | 2.87 | 15.51 | 16.36 |
| 16 | 3 | 4.50 | 39.14 | 39.43 | 15.35 | 17.50 | 17.62 | 1.59 | 13.60 | 23.18 | 26.02 |
| 17 | 2 | 1.98 | 14.08 | 13.80 | 20.82 | 22.99 | 22.99 | 0.31 | 2.18 | 11.63 | 12.62 |
| 18 | 3 | 6.71 | 30.89 | 30.86 | 22.66 | 23.87 | 23.06 | 1.93 | 8.50 | 28.54 | 28.71 |
| 19 | 2 | 1.44 | 5.82 | 5.69 | 10.54 | 10.94 | 10.94 | 0.28 | 1.11 | 7.14 | 7.37 |
| 20 | 2 | 1.31 | 7.82 | 7.43 | 5.88 | 6.94 | 6.91 | 0.24 | 1.32 | 4.72 | 5.12 |
| 21 | 3 | 31.07 | 67.38 | 66.31 | 21.40 | 24.22 | 24.70 | 10.26 | 22.31 | 27.24 | 30.91 |
| 22 | 3 | 12.82 | 2.47 | 2.53 | 35.59 | 44.12 | 44.35 | 2.67 | 0.49 | 30.32 | 35.86 |
| 23 | 1 | 0.87 | 0.83 | 0.85 | 3.74 | 3.76 | 3.67 | 1.78 | 1.76 | 19.53 | 20.21 |
| 24 | 3 | 24.53 | 0.60 | 0.56 | 24.06 | 28.76 | 28.45 | 5.30 | 0.13 | 22.74 | 26.85 |
| 25 | 3 | 1.73 | 66.99 | 66.38 | 20.54 | 30.51 | 30.53 | 0.54 | 20.94 | 29.42 | 43.49 |
| 26 | 3 | 1.27 | 28.66 | 28.95 | 16.25 | 21.29 | 21.98 | 0.37 | 8.13 | 24.57 | 31.74 |
| 27 | 1 | 1.83 | 1.83 | 1.85 | 2.43 | 2.38 | 2.41 | 7.12 | 7.41 | 34.71 | 35.37 |
| 28 | 3 | 7.74 | 14.63 | 14.63 | 4.31 | 4.42 | 4.44 | 15.20 | 29.03 | 44.26 | 45.85 |
| 29 | 1 | 6.11 | 8.80 | 8.69 | 4.47 | 4.77 | 4.71 | 2.81 | 3.95 | 11.18 | 11.66 |
| 30 | 3 | 2.00 | 44.13 | 45.13 | 9.21 | 12.15 | 12.41 | 0.83 | 17.55 | 21.67 | 27.47 |
| 31 | 3 | 7.50 | 62.30 | 57.11 | 16.17 | 18.93 | 19.08 | 2.20 | 18.18 | 23.15 | 26.95 |
| 32 | 1 | 2.40 | 2.57 | 2.59 | 1.33 | 1.40 | 1.30 | 1.15 | 1.24 | 2.99 | 3.16 |

Ca = calcium; Crea = creatinine; FE_Ca_ = fractional excretion of calcium; FE_Mg_ = fractional excretion of magnesium; Mg = magnesium

*grading of the amount of crystals, 1 = few, 2 = some, 3 = many
